# Ebolaviruses: New roles for old proteins

**DOI:** 10.1371/journal.pntd.0006349

**Published:** 2018-05-03

**Authors:** Diego Cantoni, Jeremy S. Rossman

**Affiliations:** School of Biosciences, University of Kent, Canterbury, United Kingdom; University of Texas Medical Branch, UNITED STATES

## Abstract

In 2014, the world witnessed the largest *Ebolavirus* outbreak in recorded history. The subsequent humanitarian effort spurred extensive research, significantly enhancing our understanding of ebolavirus replication and pathogenicity. The main functions of each ebolavirus protein have been studied extensively since the discovery of the virus in 1976; however, the recent expansion of ebolavirus research has led to the discovery of new protein functions. These newly discovered roles are revealing new mechanisms of virus replication and pathogenicity, whilst enhancing our understanding of the broad functions of each ebolavirus viral protein (VP). Many of these new functions appear to be unrelated to the protein’s primary function during virus replication. Such new functions range from bystander T-lymphocyte death caused by VP40-secreted exosomes to new roles for VP24 in viral particle formation. This review highlights the newly discovered roles of ebolavirus proteins in order to provide a more encompassing view of ebolavirus replication and pathogenicity.

## Author summary

Between 2014 and 2016, West Africa experienced the largest *Ebolavirus* outbreak in recorded history. The international containment effort spurred extensive research that is enhancing our understanding of ebolavirus replication and pathogenicity. Much has been learned about the main function of each ebolavirus protein since the discovery of the virus in 1976; however, recent ebolavirus research has led to the discovery of many new protein functions. These newly discovered roles are revealing new mechanisms of virus replication and pathogenesis and increasing our understanding of how each component of the virus works. This review highlights the newly discovered roles of ebolavirus proteins in order to provide a more encompassing view of ebolavirus replication and pathogenicity.

## Introduction

Ebolaviruses are negative-sense single-stranded RNA (ssRNA) viruses capable of causing acute haemorrhagic fever. The prototypical Ebola virus (EBOV; *Zaire ebolavirus*) was responsible for the recent West Africa outbreak that resulted in 28,000 cases and 11,000 deaths between 2014 and 2016 [1]. The reservoir of this lethal pathogen has not been conclusively proven, though there is good evidence suggesting fruit bats as a primary source of exposure [2]. Furthermore, survivors of ebolavirus disease (EVD) can carry the virus in immunologically privileged sites for over one year, post-recovery from the acute infection [3,4]. Resultantly, there is an increased awareness of the risk of alternate forms of transmission in EVD as survivors may carry EBOV in semen for over 12 months, causing sexual transmission, or in breast milk, which can lead to infection of newborns [4,5]. In 2004, funding from Project BioShield (United States Department of Health and Human Services) spurred research that has led to the development of novel therapeutics to prevent and treat EVD, resulting in a very promising vaccine that was evaluated during the 2014 outbreak and showed 100% efficacy in disease prevention [6,7]. Following Project BioShield and the 2014 West Africa outbreak, there has been extensive research into the ebolaviruses that has greatly expanded our understanding of viral replication and pathogenesis.

**Fig 1:**
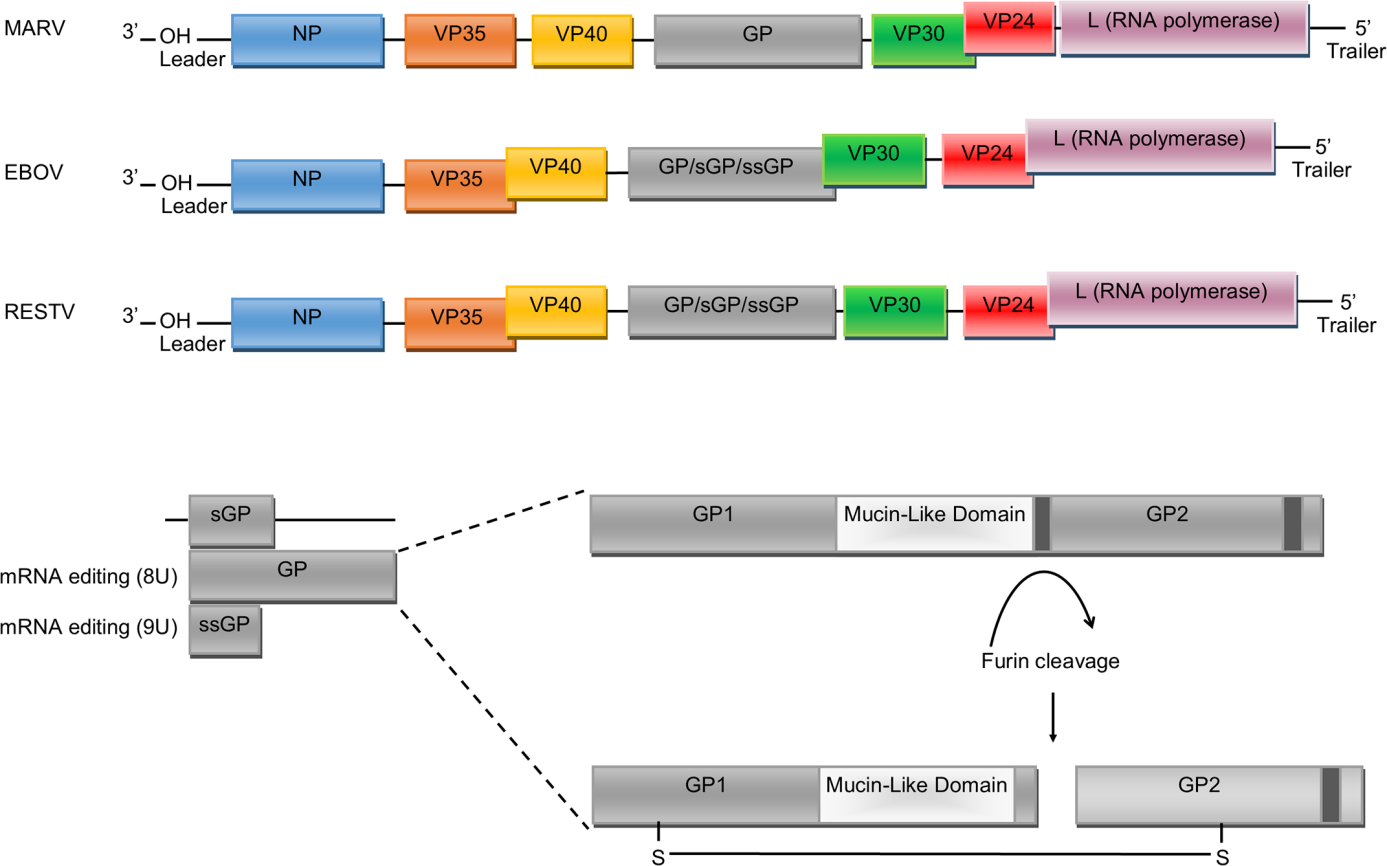
Genome organisation of Filoviridae family members: MARV, EBOV, and RESTV. Each box represents the open-reading frames that produce the VPs. RNA editing by addition of Us occurs on the GP transcript in order to produce GP and the ssGP. Post-translational modification of the GP protein results in two GP subunits after cleavage by host furin. Slight differences in gene overlaps and the presence of intergenic regions are evident between the different ebolavirus species, though the functional consequence of these differences is not clear at present. EBOV, Ebola virus; GP, glycoprotein; L, L-polymerase; MARV, Marburg virus; NP, nucleoprotein; RESTV, Reston virus; sGP; soluble glycoprotein; ssGP, small soluble glycoprotein; U, uridine; VP, viral protein.

*Ebolavirus* is a genus within the family Filoviridae, which also includes the genus *Marburgvirus* (e.g., Marburg virus: MARV) and *Cuevavirus* (e.g., Lloviu virus) [8]. The *Ebolavirus* genus contains five species: *Zaire ebolavirus*, *Sudan ebolavirus*, *Taï Forest ebolavirus*, *Bundibugyo ebolavirus*, and lastly, *Reston ebolavirus*, the only member that is nonpathogenic in humans for reasons that are still unclear [9]. The majority of research has focused on *Zaire ebolavirus*, as this species has been associated with the greatest number of outbreaks and has the highest case–fatality rates of all the ebolaviruses (30%–90%, depending on the specific outbreak), though as a consequence, many novel or species-specific functions of ebolavirus proteins may be undiscovered. The 19 kb viral genome encodes for seven main proteins: nucleoprotein (NP), glycoprotein (GP), L-polymerase (L) protein, viral protein (VP) 24, VP30, VP35, and VP40 (Fig 1) [10]. L is an RNA-dependent RNA polymerase (RdRp) and forms the RdRp complex with VP30 that is responsible for viral genome transcription and replication. VP24 and VP35 inhibit interferon (IFN) signalling and facilitate evasion of the host immune response. NP encapsidates the viral genome into the nucleocapsid, whilst VP40 drives viral assembly and budding. GP is the only protein on the surface of the virion and is essential for binding to target cells and subsequently mediating membrane fusion and the release of the viral genome. However, recent research has uncovered a multitude of new, often overlapping roles for ebolavirus proteins, and it is not possible to view viral replication as a one-protein–one-function process (Table 1). In this review, we examine the collection of recently identified secondary functions of ebolavirus proteins in order to provide a more comprehensive understanding of roles of each protein in viral replication and pathogenicity.

**Table 1:** Summary of ebolavirus protein roles.

Protein	Main Role	Secondary Role
VP24	Inhibits IFN-α/β and IFN-γ signalling through interactions with importins, STAT signalling pathways, and NF-κB signalling pathways	Nucleocapsid assembly and stability RNA incorporation into VLPs Regulates transcription and translation
VP30	Initiates ebolavirus transcription	Counters RNA interference
VP35	Inhibits type-I IFN signalling by inhibiting activation of IRF-3 via dsRNA binding Inhibits type-I IFN production by upregulating SUMOylation of IRF-7 Impairs dendritic cell maturation Counters RNA interference Inhibits antiviral effects by blocking protein kinase R	Assembly of viral complex with NP and VP30 Regulates RNA synthesis by modulating NP–RNA interactions and by interacting with dynein LC8
VP40	Viral assembly and budding	Counters RNA interference Induces apoptosis in bystander lymphocytes
GP	Virus attachment and entry	Late-stage cytotoxicity in cells Decreases endothelial barrier function Directly triggers T-lymphocyte death and augments monocyte maturation
NP	Key component of ribonucleoprotein complex, encapsidates viral genome and protects viral mRNA from degradation	
L	Involved in transcription and regulation of viral genome and mRNA editing	

**Abbreviations:** dsRNA, double-stranded RNA; GP, glycoprotein; IFN, interferon; IRF, interferon regulatory factor; L, L-polymerase; LC8, light chain 8; NF-κB, nuclear factor kappa B; NP, nucleoprotein; STAT, signal transducer and activator of transcription; SUMO, small ubiquitin-like modifier; VLPs, virus-like particles; VP, viral protein

## VP24

VP24 is one of the most studied filovirus proteins, with the majority of studies focusing on its primary role in inhibiting the host IFN response. VP24 is known to inhibit IFN-α/β and IFN-γ activation by binding to key host proteins from the karyopherin α family (karyopherin α1, α5, and α6) preventing their binding to and the subsequent nuclear import of signal transducer and activator of transcription 1 (STAT1) [11]. In addition, VP24 blocks IFN signalling by directly binding to STAT1, preventing its phosphorylation, nuclear import, and transcription of IFN-stimulated genes (ISG) [11–13]. Recent data suggest VP24 is also able to block IFN induction by supressing nuclear factor-kappa B (NF-kB) activation following tumour necrosis factor alpha (TNF-α) stimulation [14] and by supressing retinoic acid-inducible gene I (RIG-I)–dependent activation of IFN-γ1 gene expression [15]. Furthermore, the innate response antagonist domains (IRAD) in EBOV VP24 and VP35 have been implicated in preventing the maturation of EBOV-infected dendritic cells by modulating global gene expression [16,17]. This effect required both VP24 and VP35 IRAD domains, though the VP24 IRAD domain alone was sufficient to down-regulate cytokine signalling pathways [17]. These results highlight the importance of cooperative action of multiple viral proteins for evasion of the host immune response.

VP24 has also recently been seen to function in capsid assembly [18]. NP and VP35 are known to be the major components of the viral nucleocapsid, though VP24 is weakly associated and may act as a catalyst for particle formation [18]. Further evidence for a structural role of VP24 comes from the observation that N- or C-terminal deletions in VP24 inhibited the formation of nucleocapsid-like structures mediated by VP24, VP35, and NP coexpression [19]. It was suggested that the N-terminal domain of VP24 facilitates capsid formation by mediating protein–protein interactions. This is supported by the observation that mutation of the VP24 N-terminal domain results in protein aggregation [19]. A recent study confirmed the interaction between VP24 and NP, showing that VP24 residues V170 and N171 are located on a highly conserved exposed loop that interacts with NP during nucleocapsid assembly [20]. Coexpression of VP24 and VP40 results in a greater production of virus-like particles (VLPs) than when VP40 is expressed alone [21]. Similarly, in live EBOV infection, VP24 small interfering RNA (siRNA) knockdown decreases viral budding and increases the retention of viral proteins within the cell [22]. Further evidence suggests that VP24 binds to VP35 on the outer surface of the nucleocapsid where it organises the adjacent NP layer, promoting nucleocapsid stability and explaining the observed interactions between NP, VP24, and VP35 during nucleocapsid formation [23].

In addition to its role in nucleocapsid stability, VP24 may be necessary for incorporation of the viral RNA (vRNA) genome into the nucleocapsid. Several studies have shown that VP24, together with VP35, induce conformational changes in NP that are necessary for RNA encapsidation [18,24,25]. It was shown that VP24 may be directly involved in length-dependent vRNA interactions and packaging [26]. In the study, transcription- and replication-competent virus-like particles (trVLPs) were analysed for RNA content, and a significant reduction of packaged RNA was observed when VP24 was knocked down with an interfering micro-RNA (miRNA). The trVLPs that were produced showed a 2-fold reduction in RNA content and a 10-fold reduction of infectivity, suggesting that VP24 may play an essential role in RNA packaging. Furthermore, disruption of the VP24–NP interaction reduced RNA packaging and resulted in a significant reduction in reporter activity, highlighting the importance of VP24 in RNA packaging [20]. In addition, trVLP reporter gene activity was significantly affected by the presence of VP24 in a genome-length-dependent manner. Using the trVLP tetracistronic genome system, the presence of VP24 during VLP production resulted in a 25-fold increase in reporter gene activity upon subsequent infection, whereas the presence of VP24 had no effect when monocistronic minigenome systems were used in the trVLP, suggesting a genome-length-dependent role for VP24 in RNA packaging and VLP infectivity. Surprisingly, VP24 may also have a length-dependent role in transcriptional regulation. It was observed that VP24 moderately inhibited the expression of reported genes from monocistronic minigenome plasmids [27]. However, VP24 expression had no effect on protein expression from the trVLP tetracistronic genomes [26]. Whilst the impact of VP24 on protein expression is not clear, VP24 itself may be subject to length-dependent transcriptional regulation. Recent work has implicated the length of the intergenic region (IR) between VP30 and VP24 as having a significant impact on VP24 expression by regulating transcription initiation frequency (Fig 1) [28,29]. The importance of the broad range of VP24 functions during virus replication is highlighted by the fact that it has not been possible to create a VP24-deficient recombinant EBOV, even when VP24 is supplied in *trans* [22].

## VP35

**Fig 2:**
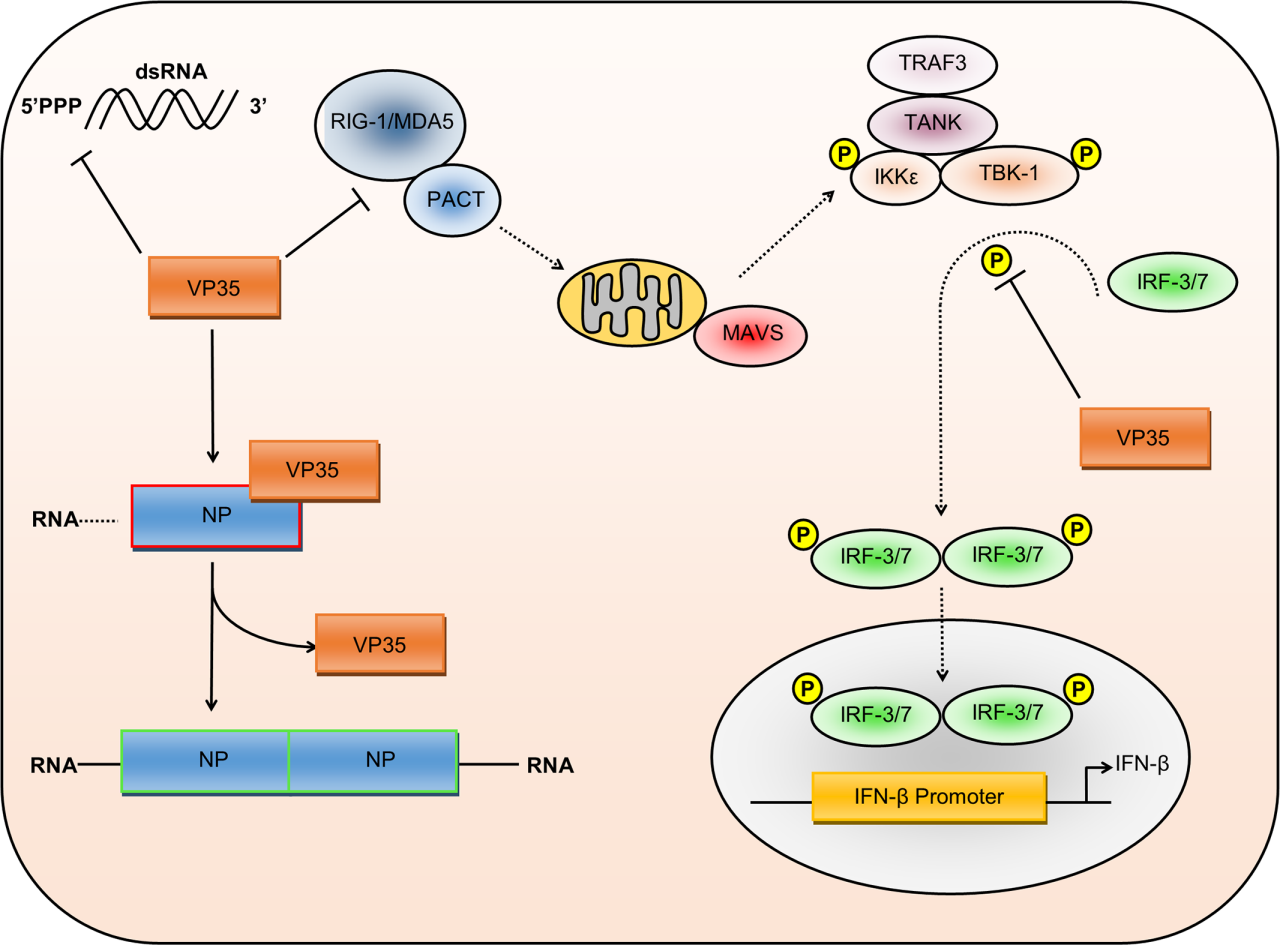
Multiple roles of VP35 during virus replication. VP35 inhibits the type-I IFN response through several different mechanisms. VP35 can bind to dsRNA, preventing the activation of RIG-I signalling. In addition, VP35 blockade of IRF3 and IRF7 phosphorylation inhibits the production of IFN-β. Recent studies have also highlighted the importance of VP35 in regulating NP–RNA association. During viral genome replication, the VP35 N-terminal peptide binds to NP, enabling the vRNA to associate with the RdRp complex for replication. During virus assembly, VP35 disassociates, enabling NP to oligomerise, bind RNA, and form the nucleocapsid. 5’PPP, 5’ triphosphate; dsRNA, double-stranded RNA; IFN, interferon; IKK, inhibitor of nuclear factor kappa B kinase subunit epsilon; IRF, interferon regulatory factor; MAVS, mitochondrial antiviral-signalling protein; MDA5, melanoma differentiation-associated protein 5; NP, nucleoprotein; PACT, protein activator of the interferon-induced protein kinase; RdRp, RNA-dependent RNA polymerase; RIG-I, retinoic acid-inducible gene I; TANK, tumour necrosis factor–receptor-associated factor family member–associated nuclear factor kappa B activator; TBK1, tumour necrosis factor–receptor-associated factor family member–associated nuclear factor kappa B activator binding kinase 1; TRAF3, tumour necrosis factor–receptor-associated factor 3; VP, viral protein; vRNA, viral RNA.

As with VP24, VP35 is primarily known for its multifaceted ability to suppress the host cell immune response. VP35 is a type-I IFN antagonist, inhibiting the activation of interferon regulatory factor (IRF)-3 via double-stranded RNA (dsRNA) binding and reducing IFN-α/β production by inhibiting RIG-I signalling [30–32]. VP35 also blocks IFN production by increasing protein inhibitor of activated STAT1 (PIAS1)-mediated SUMOylation of IRF-7, thus inhibiting IFN production following toll-like receptor (TLR) and RIG-I activation [33]. Lastly, VP35 is a suppressor of RNA silencing, functionally equivalent to the human immunodeficiency virus (HIV-1) *Trans*-activator of transcription (Tat) protein and important for viral evasion of the innate immune response [34]. Together, there is significant evidence demonstrating VP35’s intricate ability to inhibit innate immune signalling and the host antiviral response (Fig 2).

Recent work suggests that VP35 may have more diverse functions during virus replication, as VP35 was shown to interact with L and facilitate genome transcription through the formation of the RdRp complex and genome packaging through association with NP [35–37]. The first 450 residues of VP35 appear to be essential for binding to L and thus RdRp function, whereas the C-terminus associates with NP, thus linking NP and L during nucleocapsid assembly [36,38]. N-terminal deletions in VP35 block these interactions and were sufficient to inhibit the replication and transcription of an EBOV minigenome system [38]. The role of the VP35 C-terminus in capsid assembly is perhaps surprising, as this region contains the IFN inhibitory domain responsible for its main role in immune evasion. However, this domain contains several conserved stretches of basic residues involved in dsRNA binding and IFN inhibition, whereas a preceding stretch mediates interaction with NP [36]. Further research found the VP35–NP interaction controls the switch between RNA-bound NP and free NP, thus switching between genome replication and genome packaging in the nucleocapsid [39]. An N-terminal peptide derived from the VP35 NP-binding protein region (NPBP) binds NP with high affinity, causing the release of RNA from NP and resulting in the activation of genome transcription and the inhibition of NP oligomerisation (Fig 2) [39]. Additional investigation of VP35–NP binding showed two further interaction sites. Hydrophobic VP35–NP binding at these sites inhibited NP oligomerisation and prevented NP–RNA binding by blocking access to the RNA binding domain [40]. Work by Leung and colleagues suggests that during nucleocapsid formation, the NPBP peptide first disassociates from NP, then RNA binds to NP, followed by NP oligomerisation. In contrast, Kirchdoerfer and colleagues show that monomeric NP has no significant affinity for RNA, suggesting that the NPBP peptide would be displaced by an additional NP molecule, causing NP oligomerisation that would then allow for RNA binding. However, in either process, VP35–NP interactions are crucial for virus replication and are being explored as targets for future therapeutics [41,42].

VP35 also undergoes further protein–protein interactions that may affect viral genome transcription through the interaction with the cytoplasmic dynein light chain (LC8) [43]. LC8 is a highly conserved 8 kDa subunit of the cytoplasmic dynein motor complex but can also exist as a dimer in soluble form, which can affect viral transcription and assembly [44,45]. LC8 was seen to stabilise VP35 N-terminal oligomerisation in a dose-dependent manner and enhance viral genome synthesis [46]. It was noted that LC8 functions mostly in the early stages of infection, enhancing early viral gene expression before the host cells are able to establish the antiviral state. Thus, VP35 modulation of vRNA transcription can facilitate virus replication while simultaneously enhancing immune evasion.

## VP30

The minor nucleoprotein VP30 has the primary role of initiating EBOV transcription [47]. It is dynamically phosphorylated, whereby upon phosphorylation, transcription is negatively regulated, enabling binding to NP [48,49]. In turn, this permits interactions that regulate vRNA synthesis [37]. VP30 binds zinc ions due to the presence of an unconventional zinc-binding motif, facilitating RNA binding and increasing viral genome transcription [50–53].

**Fig 3:**
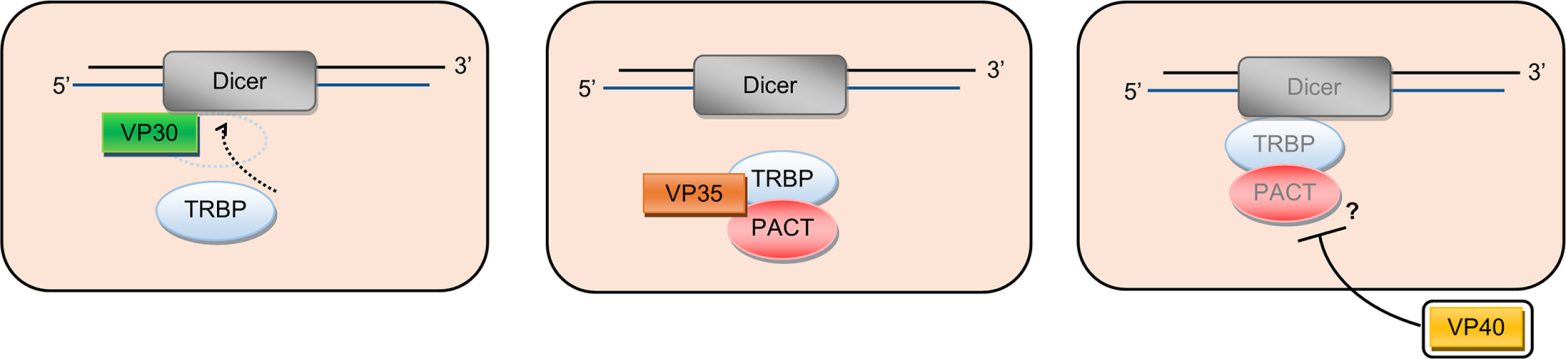
Ebolavirus proteins VP30, VP35, and VP40 are suppressors of RNA silencing. Cellular RNA interference requires the assembly of the Dicer:TRBP:PACT complex. VP30 inhibits RNAi by interacting with Dicer, preventing TRBP binding and complex activity. VP35 also inhibits complex assembly by binding TRBP and PACT, preventing their association with Dicer. VP40 suppresses RNAi during infection or when transferred to bystander immune cells through exosomes, though the mechanism by which VP40 inhibits the Dicer machinery is currently unknown. PACT, protein activator of the interferon-induced protein kinase; RNAi, RNA interference; TRBP, *Trans*-activation response RNA binding protein; VP, viral protein.

In addition to its RNA-binding role in transcription, VP30 also interferes with cellular RNA silencing [54]. In the presence of siRNA, VP30 was seen to interact with the essential RNA interference (RNAi) protein Dicer, though the VP35 N-terminus RNA binding domain was not required for the interaction or for the suppression of RNAi (Fig 3). As with the RNA silencing suppressor activity of VP35, the exact role of RNAi in antiviral immunity is not clear, nor is the consequence on EBOV replication of blocking miRNA/siRNA processing as mediated by VP30 [55]. However, despite the fact that RNA binding is not required for RNA silencing suppression, VP30 was seen to bind to a variety of nonviral RNAs. VP30–RNA binding required specific base composition and structure of the target RNA molecule [53], though it is not clear if there is a function of VP30 binding to nonviral RNA or if this is a consequence of its necessary binding to vRNA during transcription.

## VP40

The matrix protein VP40 has roles predominantly in virus assembly and budding [56,57]. VP40 can assemble either as a hexamer, which appears to be involved in budding, or as an octamer that functions in genome replication and RNA binding [58,59]. Most research has focused on the role of VP40 in assembly and budding; however, recent studies have begun to elucidate novel roles.

VP40 is known to be sufficient to mediate the formation and budding of VLPs; however, recent results have demonstrated that VP40 induces the formation for exosomes that are capable of inducing bystander cell death [60,61]. Exosome release has been seen in virally infected cells, and VP40 expression was seen to increase the expression of several endosomal sorting complex required for trafficking (ESCRT) proteins involved in exosome biogenesis, including tumour susceptibility gene 101 (TSG101), vacuolar protein-sorting-associated protein 25 (VPS25), and VPS36 [61,62]. This is consistent with previous reports showing VP40 utilising ESCRT proteins to aid viral budding, though how VP40 switches between budding and exosome release is not clear [63–65].

Transfer of VP40-induced exosomes to naïve T lymphocytes and monocytes induced apoptosis and significantly reduced cell viability similarly to that seen when exosomes from virally infected cells were used [61,62]. However, Pleet and colleagues noted that the presence of VP40 in the exosomes caused a down-regulation of miRNA machinery in both the donor and recipient cells, including a reduction in the expression of Dicer, argonaute-1, and Drosha (Fig 3). It was previously noted that VP35, VP30, and VP40 are capable of interacting with the miRNA/RNAi pathway [54]; however, this was the first demonstration that the suppression of RNA silencing can be transferred to naïve cells in the absence of virus. Currently, the precise mechanism in which VP40 interacts with miRNA machinery has yet to be characterised, and it is not yet known if the action of VP40 on the miRNA machinery directly causes apoptosis or if the exosomes contain other proteins or RNAs that may be responsible for causing the induction of apoptosis. However, the repression of key proteins in the miRNA pathway has been previously linked to the induction of apoptosis; thus, the suppression of RNA silencing may serve both to directly counteract the innate cellular immune response and to induce apoptosis of bystander immune cells, blocking the activation of adaptive immunity [57,61,66].

Due to the essential role of VP40 in viral assembly and budding, several studies have looked at inhibiting VP40 for the creation of new antiviral therapeutics [67,68]. As VP40 also plays a role in immune evasion (via RNAi suppression and exosome-bystander cell death), there is increased motivation for developing therapeutics that target one or multiple functions of VP40. It is known that viral replication requires VP40 phosphorylation at tyrosine 13 by the cellular tyrosine kinase Abelson murine leukaemia viral oncogene homologue 1 (c-Abl1) [69]. In addition, recent results showed that cyclin-dependent kinase 2 (Cdk2) in complex with Cyclin A or Cyclin E phosphorylated exosomal VP40 at serine-233 [61]. Thus, the inhibition or modulation of VP40 phosphorylation may be a target for new therapeutics. Lastly, Pleet and colleagues showed that treatment with the FDA-approved drug Oxytetracycline reduced VP40 exosome release and significantly increased donor cell viability upon treatment with VP40 exosomes, further suggesting that targeting the secondary functions of VP40 may be a new approach for developing antivirals for EVD.

## GP

The GP gene has been shown to encode for three different products due to transcriptional editing by L: full length GP, which consist of GP1 (receptor binding) and GP2 (viral fusion) subunits; soluble GP (sGP), which lacks the transmembrane domain; and small soluble GP (ssGP) [70,71]. Due to furin cleavage of sGP, a smaller cleaved fragment is also produced, called Δ-peptide [72]. GP is the only viral protein located on the surface of the virion and has a critical role in attachment and fusion [73–75]. Ebolaviruses are thought to predominantly enter cells via GP-dependent macropinocytosis, though other mechanisms have been reported, depending on factors such as host cell type [76,77]. After entry, GP directs fusion between the viral membrane and endolysosomes that contain the viral receptor Niemann-Pick C1 (NPC1) and two-pore segment channel 2 (TPC2), enabling release of the viral genome [78,79]. During the 2014–2016 outbreak, a mutation in GP at A82V was detected with high frequency [80–82]. This mutation increased GP membrane fusion activity and increased infectivity in a variety of cell types, including chimpanzee fibroblasts (S008842), rhesus epithelial (FRhK4), African green monkey epithelial (Vero), and human dendritic cells. The authors propose that this mutation is likely a result of EBOV adaptation to the human host, as several viral variants have been seen to increase human cell infectivity while decreasing virus entry in nonhuman primates [83–85]. Thus, the specific nature of protein function needs to be considered in the context of the given host.

GP has been shown to have a multitude of secondary roles beyond attachment and fusion that affect both virus replication and pathogenicity. Several studies have shown that GP contributes to EBOV virulence; however, it is not sufficient on its own to be defined as a virulence marker, despite having marked effects beyond entry and fusion [86]. EBOV GP expression has been well established as having a cytotoxic effect on host cells [87–89]. GP cytotoxicity is mediated through the mucin-like domain and its effect on the extracellular signal–regulated kinase (ERK) mitogen-activated protein kinase (MAPK) pathway [90]. EBOV GP reduces the phosphorylation and catalytic activity of ERK2, resulting in the loss of cell adherence, cell rounding, and the induction of non-apoptotic cell death. The decrease in ERK2 activity was also necessary for GP-induced down-regulation of αV integrin expression, further impairing cell adherence and tight junction formation. In addition, the sGP cleavage product, Δ-peptide, may play a role in EBOV pathogenicity by acting as a viroporin [91]. Δ-peptide is able to form pores in the plasma membrane of mammalian cells, increasing ion permeability and causing cytotoxicity [92]. However, it is not known if the Δ-peptide can be released from cells or if its induction of cytotoxicity is limited to within the infected cell. In order to regulate the toxicity caused by GP and its cleavage products at early stages of infection, GP expression is dynamically regulated [93]. The balance and timing of EBOV GP/sGP/ssGP/shed-GP/Δ-peptide expression has been found to be pivotal in virus replication, affecting not only cell death but viral assembly and budding [94].

Whilst VP40 expression is sufficient to produce VLPs, GP expression enhances VLP generation, suggesting a possible secondary role for GP in viral egress [21]. Recent work suggests that GP does not directly affect viral assembly or budding, but rather counteracts the cellular budding restriction factor tetherin [95]. In the absence of GP, VLPs assemble and bud but are retained on the cell surface through the antiviral tethering actions of the tetherin protein. GP expression enabled VLP release but did not affect tetherin cell-surface localisation, nor was a specific GP–tetherin interaction found [96]. It was shown that the GP glycosylation and its receptor-binding domain (RBD) were critical for anti-tetherin activity, though mutation of the RBD did not affect interactions with tetherin, and inhibition of GP–NPC1 binding did not affect the anti-tetherin activity [97]. Instead, it is thought that GP is specifically able to block the association between VP40 and tetherin, though the nature of tetherin–VP40 interaction and the mechanism of GP inhibition is not known [98].

EBOV infection causes significant impairment of the endothelial barrier function. GP repression of ERK2 activity reduces integrin expression and cell adherence; however, GP also induces endothelial cell activation, further decreasing endothelial barrier functions [99]. During EBOV infection of endothelial cells, cell adhesion molecules (CAM) ICAM-1 and VCAM-1 were found to be transcriptionally upregulated, with increased cell surface expression of CAMs. The activity occurs with cellular GP expression as well as following the transfer of GP-containing VLPs; viral replication does not appear to be required. Endothelial activation was not observed in the absence of GP, nor with the GP transcriptional variant sGP or the GP cleavage product Δ-peptide [99]. GP-induced endothelial cell activation may facilitate decreased barrier function, whilst the up-regulation of CAMs may facilitate adhesion and subsequent infection of immune cells, such as macrophages. A model was proposed whereby activated endothelial cells result in increased leukocyte recruitment, resulting in thrombomodulin release, resulting in an activated, leukocyte-rich endothelium in a procoagulant state [100]. Whether this is an unintended consequence of GP or has a role in increased viral spread and infectivity is yet to be investigated.

EBOV GP has also been implicated in modulation of the host immune system. GP on the cell plasma membrane has been shown to be cleaved by TNF-α-converting enzyme (TACE), resulting in the release of a soluble cleaved product called shed GP that is missing the transmembrane domain [101]. Shed GP was seen to activate uninfected macrophages and dendritic cells, resulting in the production of multiple pro- and anti-inflammatory cytokines, including TNF-α, with subsequent effects on vascular permeability [102]. It is thought that shed GP activates macrophages by binding to and activating toll-like receptor 4 (TLR4) in a manner requiring GP glycosylation. Recently it was also shown that full-length GP on VLPs can also trigger the activation of TLR4 in macrophages, resulting in a similar activation phenotype [103]. In contrast, GP binding to TLR4 on T lymphocytes directly triggers cell death through an up-regulation of caspase 9, even in the absence of infection [104]. In dendritic cells, GP was found to interact with the liver and lymph node sinusoidal endothelial cell C-type lectin (LSECtin), a C-type lectin that contains two amino acids, residues N256 and N274, that bind GP in a Ca^2+^-dependant manner, triggering the activation of spleen tyrosine kinase (Syk) signalling and the production of inflammatory cytokines TNF-α and interleukin-6 (IL-6) [105]. As shed GP retains most of the structure of full-length GP, this soluble molecule is capable of binding to and neutralising circulating anti-GP antibodies, facilitating viral immune evasion [101].

Similarly, sGP has been implicated in evading the immune system via antigenic subversion [106]. It was found that boosting GP-immunised mice with sGP biased B-cell response towards epitopes that were shared between sGP and GP, reducing GP-specific antibody production and possibly impeding the immune-mediated clearance of EBOV infection. The structure of sGP in complex with antibodies was recently solved and highlights differences in antibody reactivity between GP and sGP [107]. Whilst GP is trimeric, sGP oligomerises into a parallel homodimer. Cross-reactive c13C6 antibody epitopes are presented similarly on GP and sGP, though the authors report that one c13C6 antibody binds to one GP trimer, whereas multiple different immune-complexes were formed with sGP, ranging from rectangular complexes with a 2:2 ratio of c13C6 antibody to sGP dimer up to pentagonal 5:5 antibody:sGP complexes [107]. This further supports the hypothesis that sGP enhances viral immune evasion by biasing the antibody response towards sGP binding.

Whilst the multitude of GP secondary effects have a significant impact on EBOV infection, pathogenesis, and immune evasion, GP remains the immunodominant protein on the EBOV virion, and vaccination with the GP protein on pseudotyped viruses, recombinant vesicular stomatitis virus with *Zaire ebolavirus* GP (rVSV-ZEBOV), has been highly effective in preventing EVD [7].

## NP and L

NP has a distinct function in the replication cycle as it is a key component of the viral ribonucleoprotein complex and has critical roles in protecting vRNA from degradation and in mediating genome encapsidation during virus assembly [10]. At present, all research has focused on these primary activities of NP, and any secondary roles remain to be determined [18,25,40]. Similarly, the RNA-dependent L-polymerase is an essential component of the RdRp complex and required for viral genome transcription and replication [10]. It has been observed that L can also edit mRNA, as seen with the GP gene, where L-editing results in the production of the GP transcript instead of sGP [108]. L-editing may also regulate the different expression levels of GP, sGP and ssGP. During serial passage in tissue culture cells, L was found to add a single uridine (U) residue to a site consisting of 7 Us in the GP gene, changing the expression ratio of GP:sGP to 80:20. A single passage in guinea pigs caused reversion of the genome back to 7 Us and changed the GP:sGP expression ratio back to 20:80, which may facilitate immune evasion during in vivo replication [109]. In contrast, during viral replication in the human hepatocarcinoma cell line (Huh7), a 9U variant was seen that retained the high level expression of sGP but had enhanced expression of ssGP [71]. It is speculated that these rapid alterations in the GP gene may act as a regulatory mechanism, enabling efficient virus replication in different host environments. At present, no other roles for the L protein have been postulated.

## Conclusion

For many years now, the fundamental principles of ebolavirus replication have been known, with each viral protein playing a specific role: L and VP30 form the RdRp and mediate viral genome transcription and replication, NP packages the vRNA genome and forms the nucleocapsid, VP40 mediates virion assembly and budding, GP mediates virion attachment to and fusion with the host cell, and VP24 and VP35 enable evasion of the host immune system. However, in recent years, our understanding of ebolavirus replication and pathogenesis has significantly increased, and we now know that each viral protein plays a multitude of overlapping roles during virus replication.

VP24 and VP35 were thought to primarily mediate immune evasion but have now been seen to function in viral replication and in the formation of the nucleocapsid. Similarly, many of the viral proteins that were thought to play functional roles in replication (i.e., VP30, VP40, GP) have now been discovered to have significant roles in immune evasion. This broadens our understanding of the ways in which ebolaviruses can evade and subvert the host immune system. Of particular interest is the observation that VP30, VP35, and VP40 all appear to supress the host RNA silencing system, though the relevance of this to viral replication is not yet clear (Fig 3). In addition, many ebolavirus proteins are seen to interact with immune cells, causing cell activation and/or cell death and facilitating both viral replication and spread (e.g., by recruiting monocytes to infected cells or by increasing vascular leakage) as well as enabling immune evasion (e.g., antibody neutralisation by sGP and by cleaved GP), roles that were previously solely attributed to VP24 and VP35.

Since Project BioShield was initiated in 2004, there has been a substantial increase in our understanding of ebolavirus replication and pathogenesis. Several vaccines and antiviral therapeutics have been developed and were used during the 2014–2016 West Africa Ebola virus outbreak. However, the outbreak was difficult to contain and highlighted the fact that there are many significant aspects of EBOV that we do not yet understand (e.g., EBOV persistence in immunologically privileged tissue) and that there is a pressing need for continued research to better understand and combat this deadly pathogen.

**Key learning points**

- Recent research has uncovered new functions of many ebolavirus proteins that appear to be unrelated to the protein’s primary function during virus replication.
- Viral structural proteins, such as VP40 and GP, have been found to cause cell death in a wide range of cell types, including bystander T lymphocytes.
- Viral immune modulating proteins, such as VP24 and VP35, now have significantly expanded functions during virus replication, including the regulation of viral particle formation.

**Top five papers**

1. Haasnoot J, De Vries W, Geutjes EJ, Prins M, De Haan P, Berkhout B. The ebola virus VP35 protein is a suppressor of RNA silencing. PLoS Pathog. 2007;3(6):e86.
2. Leung DW, Borek D, Luthra P, Binning JM, Anantpadma M, Liu G, et al. An intrinsically disordered peptide from ebola virus VP35 controls viral RNA synthesis by modulating nucleoprotein-RNA interactions. Cell Rep. 2015;11(3):376–389.
3. Pleet ML, Mathiesen A, DeMarino C, Akpamagbo YA, Barclay RA, Schwab A, et al. Ebola VP40 in Exosomes Can Cause Immune Cell Dysfunction. Front Microbiol. 2016;7:1765.
4. Watt, A., Moukambi, F., Banadyga, L., Groseth, A., Callison, J., Herwig, A., Ebihara, H., Feldmann, H., and Hoenen, T. (2014). A novel life cycle modeling system for Ebola virus shows a genome length-dependent role of VP24 in virus infectivity. J Virol. 2014;88(18):10511–10524.
5. Yang ZY, Duckers HJ, Sullivan NJ, Sanchez A, Nabel EG, Nabel GJ. Identification of the Ebola virus glycoprotein as the main viral determinant of vascular cell cytotoxicity and injury. Nat Med. 2000;6(8):886–889.

## References

[1] WHO Ebola Response Team. After Ebola in West Africa—Unpredictable Risks, Preventable Epidemics. N Engl J Med. 2016;375(6): 587–596. doi: 10.1056/NEJMsr1513109 27509108 10.1056/NEJMsr1513109

[2] Leroy EM, Kumulungui B, Pourrut X, Rouquet P, Hassanin A, Yaba P, et al Fruit bats as reservoirs of Ebola virus. Nature. 2005;438(7068): 575–576. doi: 10.1038/438575a 16319873 10.1038/438575a

[3] Rodriguez LL, De Roo a, Guimard Y, Trappier SG, Sanchez a, Bressler D, et al Persistence and genetic stability of Ebola virus during the outbreak in Kikwit, Democratic Republic of the Congo, 1995. J Infect Dis. 1999;179 (Suppl 1): S170–S176. doi: 10.1086/514291 9988181 10.1086/514291

[4] Deen GF, Knust B, Broutet N, Sesay FR, Formenty P, Ross C, et al Ebola RNA Persistence in Semen of Ebola Virus Disease Survivors—Final Report. N Engl J Med. 2017;377: 1428–1437. doi: 10.1056/NEJMoa1511410 26465681 10.1056/NEJMoa1511410 PMC5798881

[5] Nordenstedt H, Bah EI, de la Vega MD, Barry M, N’Faly M, Barry M, et al Ebola Virus in Breast Milk. Emerg Infect Dis. 2016;22(4): 759–760. doi: 10.3201/eid2204.151880 26982461 10.3201/eid2204.151880 PMC4806941

[6] Henao-Restrepo AM, Longini IM, Egger M, Dean NE, Edmunds WJ, Camacho A, et al Efficacy and effectiveness of an rVSV-vectored vaccine expressing Ebola surface glycoprotein: interim results from the Guinea ring vaccination cluster-randomised trial. Lancet. 2015;386(9996): 857–866. doi: 10.1016/S0140-6736(15)61117-5 26248676 10.1016/S0140-6736(15)61117-5

[7] Henao-Restrepo AM, Camacho A, Longini IM, Watson CH, Edmunds WJ, Egger M, et al Efficacy and effectiveness of an rVSV-vectored vaccine in preventing Ebola virus disease: final results from the Guinea ring vaccination, open-label, cluster-randomised trial (Ebola {Ç}a Suffit!). Lancet. Elsevier; 2016; 389(10068): 505–518. doi: 10.1016/S0140-6736(16)32621-6 28017403 10.1016/S0140-6736(16)32621-6 PMC5364328

[8] Kuhn JH, Becker S, Ebihara H, Geisbert TW, Johnson KM, Kawaoka Y, et al Proposal for a revised taxonomy of the family Filoviridae: Classification, names of taxa and viruses, and virus abbreviations. Arch Virol. 2010;155(12): 2083–2103. doi: 10.1007/s00705-010-0814-x 21046175 10.1007/s00705-010-0814-x PMC3074192

[9] Cantoni D, Hamlet A, Michaelis M, Wass MN, Rossman JS. Risks Posed by Reston, the Forgotten Ebolavirus. mSphere. 2016;1: 1–10. doi: 10.1128/mSphere.00322-16 28066813 10.1128/mSphere.00322-16 PMC5196033

[10] Baseler L, Chertow DS, Johnson KM, Feldmann H, Morens DM. The Pathogenesis of Ebola Virus Disease. Annu Rev Pathol Mech Dis. 2017;12: 387–418. doi: 10.1146/annurev-pathol-052016-100506 27959626 10.1146/annurev-pathol-052016-100506

[11] Reid SP, Leung LW, Hartman AL, Martinez O, Shaw ML, Carbonnelle C, et al Ebola virus VP24 binds karyopherin alpha1 and blocks STAT1 nuclear accumulation. J Virol. 2006;80(11): 5156–67. doi: 10.1128/JVI.02349-05 16698996 10.1128/JVI.02349-05 PMC1472181

[12] Zhang APP, Bornholdt ZA, Liu T, Abelson DM, Lee DE, Li S, et al The ebola virus interferon antagonist VP24 directly binds STAT1 and has a novel, pyramidal fold. PLoS Pathog. 2012;8: e1002550 doi: 10.1371/journal.ppat.1002550 22383882 10.1371/journal.ppat.1002550 PMC3285596

[13] Xu W, Edwards MR, Borek DM, Feagins AR, Mittal A, Alinger JB, et al Ebola virus VP24 targets a unique NLS binding site on karyopherin alpha 5 to selectively compete with nuclear import of phosphorylated STAT1. Cell Host Microbe. 2014;16(2): 187–200. doi: 10.1016/j.chom.2014.07.008 25121748 10.1016/j.chom.2014.07.008 PMC4188415

[14] Guito JC, Albariño CG, Chakrabarti AK, Towner JS. Novel activities by ebolavirus and marburgvirus interferon antagonists revealed using a standardized in vitro reporter system. Virology. 2017;501: 147–165. doi: 10.1016/j.virol.2016.11.015 27930961 10.1016/j.virol.2016.11.015 PMC11524407

[15] He F, Melén K, Maljanen S, Lundberg R, Jiang M, Österlund P, et al Ebolavirus protein VP24 interferes with innate immune responses by inhibiting interferon-λ1 gene expression. Virology. 2017;509: 23–34. doi: 10.1016/j.virol.2017.06.002 28595092 10.1016/j.virol.2017.06.002

[16] Lubaki NM, Ilinykh P, Pietzsch C, Tigabu B, Freiberg AN, Koup RA, et al The lack of maturation of Ebola virus-infected dendritic cells results from the cooperative effect of at least two viral domains. J Virol. 2013;87(13): 7471–85. doi: 10.1128/JVI.03316-12 23616668 10.1128/JVI.03316-12 PMC3700277

[17] Ilinykh PA, Lubaki NM, Widen SG, Renn LA, Theisen TC, Rabin RL, et al Different Temporal Effects of Ebola Virus VP35 and VP24 Proteins on Global Gene Expression in Human Dendritic Cells. J Virol. 2015;89(15): 7567–83. doi: 10.1128/JVI.00924-15 25972536 10.1128/JVI.00924-15 PMC4505630

[18] Huang Y, Xu L, Sun Y, Nabel GJ. The assembly of Ebola virus nucleocapsid requires virion-associated proteins 35 and 24 and posttranslational modification of nucleoprotein. Mol Cell. 2002;10(2): 307–316. doi: 10.1016/S1097-2765(02)00588-9 12191476 10.1016/s1097-2765(02)00588-9

[19] Han Z, Boshra H, Sunyer JO, Zwiers SH, Paragas J, Harty RN. Biochemical and Functional Characterization of the Ebola Virus VP24 Protein: Implications for a Role in Virus Assembly and Budding. J Virol. 2003;77(3): 1793–1800. doi: 10.1128/JVI.77.3.1793-1800.2003 12525613 10.1128/JVI.77.3.1793-1800.2003 PMC140957

[20] Banadyga L, Hoenen T, Ambroggio X, Dunham E, Groseth A, Ebihara H. Ebola virus VP24 interacts with NP to facilitate nucleocapsid assembly and genome packaging. Sci Rep. 2017;7: 1–14. doi: 10.1038/s41598-016-0028-x 28794491 10.1038/s41598-017-08167-8 PMC5550494

[21] Licata JM, Johnson RF, Han Z, Harty RN. Contribution of Ebola Virus Glycoprotein, Nucleoprotein, and VP24 to Budding of VP40 Virus-Like Particles. J Virol. 2004;78(14): 7344–7351. doi: 10.1128/JVI.78.14.7344-7351.2004 15220407 10.1128/JVI.78.14.7344-7351.2004 PMC434112

[22] Mateo M, Carbonnelle C, Martinez MJ, Reynard O, Page A, Volchkova VA, et al Knockdown of Ebola virus VP24 impairs viral nucleocapsid assembly and prevents virus replication. J Infect Dis. 2011;204(Suppl 3): S892–S896. doi: 10.1093/infdis/jir311 21987766 10.1093/infdis/jir311

[23] Beniac DR, Melito PL, DeVarennes SL, Hiebert SL, Rabb MJ, Lamboo LL, et al The organisation of Ebola virus reveals a capacity for extensive, modular polyploidy. PLoS ONE. 2012;7: e29608 doi: 10.1371/journal.pone.0029608 22247782 10.1371/journal.pone.0029608 PMC3256159

[24] Noda T, Aoyama K, Sagara H, Kida H, Kawaoka Y. Nucleocapsid-like structures of Ebola virus reconstructed using electron tomography. J Vet Med Sci. 2005;67(3): 325–8. doi: 10.1292/jvms.67.325 15805739 10.1292/jvms.67.325

[25] Watanabe S, Noda T, Kawaoka Y. Functional mapping of the nucleoprotein of Ebola virus. J Virol. 2006;80(8): 3743–51. doi: 10.1128/JVI.80.8.3743-3751.2006 16571791 10.1128/JVI.80.8.3743-3751.2006 PMC1440433

[26] Watt A, Moukambi F, Banadyga L, Groseth A, Callison J, Herwig A, et al A novel life cycle modeling system for Ebola virus shows a genome length-dependent role of VP24 in virus infectivity. J Virol. 2014;88(18): 10511–24. doi: 10.1128/JVI.01272-14 24965473 10.1128/JVI.01272-14 PMC4178905

[27] Watanabe S, Noda T, Halfmann P, Jasenosky L, Kawaoka Y. Ebola virus (EBOV) VP24 inhibits transcription and replication of the EBOV genome. J Infect Dis. 2007;196 (Suppl 2): S284–S290. doi: 10.1086/520582 17940962 10.1086/520582

[28] Brauburger K, Boehmann Y, Tsuda Y, Hoenen T, Olejnik J, Schümann M, et al Analysis of the highly diverse gene borders in Ebola virus reveals a distinct mechanism of transcriptional regulation. J Virol. 2014;88(21): 12558–71. doi: 10.1128/JVI.01863-14 25142600 10.1128/JVI.01863-14 PMC4248908

[29] Neumann G, Watanabe S, Kawaoka Y. Characterization of Ebolavirus regulatory genomic regions. Virus Res. 2009;144(1–2): 1–7. doi: 10.1016/j.virusres.2009.02.005 19481829 10.1016/j.virusres.2009.02.005 PMC2845284

[30] Basler CF, Wang X, Mühlberger E, Volchkov V, Paragas J, Klenk HD, et al The Ebola virus VP35 protein functions as a type I IFN antagonist. Proc Natl Acad Sci U S A. 2000;97(22): 12289–12294. doi: 10.1073/pnas.220398297 11027311 10.1073/pnas.220398297 PMC17334

[31] Cárdenas WB, Loo Y-M, Gale M, Hartman AL, Kimberlin CR, Martínez-Sobrido L, et al Ebola virus VP35 protein binds double-stranded RNA and inhibits alpha/beta interferon production induced by RIG-I signaling. J Virol. 2006;80(11): 5168–5178. doi: 10.1128/JVI.02199-05 16698997 10.1128/JVI.02199-05 PMC1472134

[32] Luthra P, Ramanan P, Mire CE, Weisend C, Tsuda Y, Yen B, et al Mutual antagonism between the Ebola virus VP35 protein and the RIG-I activator PACT determines infection outcome. Cell Host Microbe. 2013;14(1): 74–84. doi: 10.1016/j.chom.2013.06.010 23870315 10.1016/j.chom.2013.06.010 PMC3875338

[33] Chang TH, Kubota T, Matsuoka M, Jones S, Bradfute SB, Bray M, et al Ebola Zaire virus blocks type I interferon production by exploiting the host SUMO modification machinery. PLoS Pathog. 2009;5(6): e1000493 doi: 10.1371/journal.ppat.1000493 19557165 10.1371/journal.ppat.1000493 PMC2696038

[34] Haasnoot J, De Vries W, Geutjes EJ, Prins M, De Haan P, Berkhout B. The ebola virus VP35 protein is a suppressor of RNA silencing. PLoS Pathog. 2007;3(6): e86 doi: 10.1371/journal.ppat.0030086 17590081 10.1371/journal.ppat.0030086 PMC1894824

[35] Groseth A, Charton JE, Sauerborn M, Feldmann F, Jones SM, Hoenen T, et al The Ebola virus ribonucleoprotein complex: A novel VP30-L interaction identified. Virus Res. 2009;140(1–2): 8–14. doi: 10.1016/j.virusres.2008.10.017 19041915 10.1016/j.virusres.2008.10.017 PMC3398801

[36] Prins KC, Binning JM, Shabman RS, Leung DW, Amarasinghe GK, Basler CF. Basic Residues within the Ebolavirus VP35 Protein Are Required for Its Viral Polymerase Cofactor Function. J Virol. 2010;84(20): 10581–10591. doi: 10.1128/JVI.00925-10 20686031 10.1128/JVI.00925-10 PMC2950600

[37] Kirchdoerfer RN, Moyer CL, Abelson DM, Saphire EO. The Ebola Virus VP30-NP Interaction Is a Regulator of Viral RNA Synthesis. PLoS Pathog. 2016;12(10): e1005937 doi: 10.1371/journal.ppat.1005937 27755595 10.1371/journal.ppat.1005937 PMC5068707

[38] Trunschke M, Conrad D, Enterlein S, Olejnik J, Brauburger K, Mühlberger E. The L-VP35 and L-L interaction domains reside in the amino terminus of the Ebola virus L protein and are potential targets for antivirals. Virology. 2013;441(2): 135–145. doi: 10.1016/j.virol.2013.03.013 23582637 10.1016/j.virol.2013.03.013 PMC3773471

[39] Leung DW, Borek D, Luthra P, Binning JM, Anantpadma M, Liu G, et al An intrinsically disordered peptide from ebola virus VP35 controls viral RNA synthesis by modulating nucleoprotein-RNA interactions. Cell Rep. 2015;11(3): 376–389. doi: 10.1016/j.celrep.2015.03.034 25865894 10.1016/j.celrep.2015.03.034 PMC4599368

[40] Kirchdoerfer RN, Abelson DM, Li S, Wood MR. Assembly of the Ebola Virus Nucleoprotein from a Chaperoned VP35 Complex. Cell Rep. 2015;12(1): 140–149. doi: 10.1016/j.celrep.2015.06.003 26119732 10.1016/j.celrep.2015.06.003 PMC4500542

[41] Ren JX, Zhang RT, Zhang H, Cao XS, Liu LK, Xie Y. Identification of novel VP35 inhibitors: Virtual screening driven new scaffolds. Biomed Pharmacother. 2016;84: 199–207. doi: 10.1016/j.biopha.2016.09.034 27657828 10.1016/j.biopha.2016.09.034

[42] Liu G, Nash PJ, Johnson B, Pietzsch C, Ilagan MG, Bukreyev A, et al A Sensitive In Vitro High-Throughput Screen to Identify Pan-Filoviral Replication Inhibitors Targeting the VP35-NP Interface. ACS Infect Dis. 2017;3(3): 190–198. doi: 10.1021/acsinfecdis.6b00209 28152588 10.1021/acsinfecdis.6b00209 PMC5735849

[43] Kubota T, Matsuoka M, Chang T-H, Bray M, Jones S, Tashiro M, et al Ebolavirus VP35 Interacts with the Cytoplasmic Dynein Light Chain 8. J Virol. 2009;83(13): 6952–6956. doi: 10.1128/JVI.00480-09 19403681 10.1128/JVI.00480-09 PMC2698516

[44] Kirkham JK, Park SH, Nguyen TN, Lee JH, Günzl A. Dynein Light Chain LC8 Is Required for RNA Polymerase I-Mediated Transcription in *Trypanosoma brucei*, Facilitating Assembly and Promoter Binding of Class I Transcription Factor A. Mol Cell Biol. 2016;36(1): 95–107. doi: 10.1128/MCB.00705-15 26459761 10.1128/MCB.00705-15 PMC4702606

[45] Tan GS, Preuss MAR, Williams JC, Schnell MJ. The dynein light chain 8 binding motif of rabies virus phosphoprotein promotes efficient viral transcription. Proc Natl Acad Sci U S A. 2007;104(17): 7229–34. doi: 10.1073/pnas.0701397104 17438267 10.1073/pnas.0701397104 PMC1855364

[46] Luthra P, Jordan DS, Leung DW, Amarasinghe GK, Basler CF. Ebola virus VP35 interaction with dynein LC8 regulates viral RNA synthesis. J Virol. 2015;89(9): 5148–53. doi: 10.1128/JVI.03652-14 25741013 10.1128/JVI.03652-14 PMC4403485

[47] Weik M, Modrof J, Klenk H-D, Becker S, Mühlberger E. Ebola Virus VP30-Mediated Transcription Is Regulated by RNA Secondary Structure Formation. J Virol. 2002;76(17): 8532–8539. doi: 10.1128/JVI.76.17.8532-8539.2002 12163572 10.1128/JVI.76.17.8532-8539.2002 PMC136988

[48] Modrof J, Mühlberger E, Klenk HD, Becker S. Phosphorylation of VP30 impairs Ebola virus transcription. J Biol Chem. 2002;277(36): 33099–33104. doi: 10.1074/jbc.M203775200 12052831 10.1074/jbc.M203775200

[49] Biedenkopf N, Lier C, Becker S. Dynamic Phosphorylation of VP30 is essential for Ebola virus life cycle. J Virol. 2016;90(10): 4914–4925. doi: 10.1128/JVI.03257-15 26937028 10.1128/JVI.03257-15 PMC4859730

[50] Biedenkopf N, Schlereth J, Grünweller A, Becker S, Hartmann RK. RNA-binding of Ebola virus VP30 is essential for activating viral transcription. J Virol. 2016;90(16): 7481–7496. doi: 10.1128/JVI.00271-16 27279615 10.1128/JVI.00271-16 PMC4984652

[51] John SP, Wang T, Steffen S, Longhi S, Schmaljohn CS, Jonsson CB. Ebola virus VP30 is an RNA binding protein. J Virol. 2007;81(17): 8967–8976. doi: 10.1128/JVI.02523-06 17567691 10.1128/JVI.02523-06 PMC1951390

[52] Modrof J, Becker S, Mühlberger E. Ebola Virus Transcription Activator VP30 Is a Zinc-Binding Protein. J Virol. 2003;77(5): 3334–3338. doi: 10.1128/JVI.77.5.3334-3338.2003 12584359 10.1128/JVI.77.5.3334-3338.2003 PMC149768

[53] Schlereth J, Grünweller A, Biedenkopf N, Becker S, Hartmann RK. RNA binding specificity of Ebola virus transcription factor VP30. RNA Biol. 2016;13(9): 783–98. doi: 10.1080/15476286.2016.1194160 27315567 10.1080/15476286.2016.1194160 PMC5013996

[54] Fabozzi G, Nabel CS, Dolan M a, Sullivan NJ. Ebolavirus proteins suppress the effects of small interfering RNA by direct interaction with the mammalian RNA interference pathway. J Virol. 2011;85(6): 2512–2523. doi: 10.1128/JVI.01160-10 21228243 10.1128/JVI.01160-10 PMC3067942

[55] Li Y, Lu J, Han Y, Fan X, Ding SW. RNA interference functions as an antiviral immunity mechanism in mammals. Science. 2013;342(6155): 231–234. doi: 10.1126/science.1241911 24115437 10.1126/science.1241911 PMC3875315

[56] Ruigrok RW, Schoehn G, Dessen a, Forest E, Volchkov V, Dolnik O, et al Structural characterization and membrane binding properties of the matrix protein VP40 of Ebola virus. J Mol Biol. 2000;300(1): 103–112. doi: 10.1006/jmbi.2000.3822 10864502 10.1006/jmbi.2000.3822

[57] Pleet ML, DeMarino C, Lepene B, Aman MJ, Kashanchi F. The Role of Exosomal VP40 in Ebola Virus Disease. DNA Cell Biol. 2017;36(4): 243–248. doi: 10.1089/dna.2017.3639 28177658 10.1089/dna.2017.3639 PMC5385446

[58] Timmins J, Schoehn G, Kohlhaas C, Klenk HD, Ruigrok RWH, Weissenhorn W. Oligomerization and polymerization of the filovirus matrix protein VP40. Virology. 2003;312(2): 359–368. doi: 10.1016/S0042-6822(03)00260-5 12919741 10.1016/s0042-6822(03)00260-5

[59] Bornholdt ZA, Noda T, Abelson DM, Halfmann P, Wood MR, Kawaoka Y, et al Structural rearrangement of ebola virus vp40 begets multiple functions in the virus life cycle. Cell. 2013;154(4): 763–774. doi: 10.1016/j.cell.2013.07.015 23953110 10.1016/j.cell.2013.07.015 PMC4138722

[60] Harty RN, Brown ME, Wang G, Huibregtse J, Hayes FP. A PPxY motif within the VP40 protein of Ebola virus interacts physically and functionally with a ubiquitin ligase: Implications for filovirus budding. Proc Natl Acad Sci U S A. 2000;97(25): 13871–13876. doi: 10.1073/pnas.250277297 11095724 10.1073/pnas.250277297 PMC17668

[61] Pleet ML, Mathiesen A, DeMarino C, Akpamagbo YA, Barclay RA, Schwab A, et al Ebola VP40 in Exosomes Can Cause Immune Cell Dysfunction. Front Microbiol. 2016;7: 1765 doi: 10.3389/fmicb.2016.01765 27872619 10.3389/fmicb.2016.01765 PMC5098130

[62] Wauquier N, Becquart P, Padilla C, Baize S, Leroy EM. Human fatal zaire ebola virus infection is associated with an aberrant innate immunity and with massive lymphocyte apoptosis. PLoS Negl Trop Dis. 2010;4(10): e837 doi: 10.1371/journal.pntd.0000837 20957152 10.1371/journal.pntd.0000837 PMC2950153

[63] Licata JM, Simpson-Holley M, Wright NT, Han Z, Paragas J, Harty RN. Overlapping motifs (PTAP and PPEY) within the Ebola virus VP40 protein function independently as late budding domains: involvement of host proteins TSG101 and VPS-4. J Virol. 2003;77(3): 1812–1819. doi: 10.1128/JVI.77.3.1812-1819.2003 12525615 10.1128/JVI.77.3.1812-1819.2003 PMC140960

[64] Timmins J, Schoehn G, Ricard-Blum S, Scianimanico S, Vernet T, Ruigrok RWH, et al Ebola virus matrix protein VP40 interaction with human cellular factors Tsg101 and Nedd4. J Mol Biol. 2003;326(2): 493–502. doi: 10.1016/S0022-2836(02)01406-7 12559917 10.1016/s0022-2836(02)01406-7

[65] Han Z, Madara JJ, Liu Y, Liu W, Ruthel G, Freedman BD, et al ALIX Rescues Budding of a Double PTAP/PPEY L-Domain Deletion Mutant of Ebola VP40: A Role for ALIX in Ebola Virus Egress. J Infect Dis. 2015;212(Suppl 2): S138–S145. doi: 10.1093/infdis/jiu838 25786915 10.1093/infdis/jiu838 PMC4564528

[66] Han Y, Liu Y, Gui Y, Cai Z. Inducing cell proliferation inhibition and apoptosis via silencing Dicer, Drosha, and Exportin 5 in urothelial carcinoma of the bladder. J Surg Oncol. 2013;107(2): 201–205. doi: 10.1002/jso.23214 22766726 10.1002/jso.23214

[67] Stahelin R V. Could the Ebola virus matrix protein VP40 be a drug target? Expert Opin Ther Targets. 2014;18(2): 115–20. doi: 10.1517/14728222.2014.863877 24283270 10.1517/14728222.2014.863877 PMC5543415

[68] Biedenkopf N, Lange-Grünweller K, Schulte FW, Weißer A, Müller C, Becker D, et al The natural compound silvestrol is a potent inhibitor of Ebola virus replication. Antiviral Res. 2017;137: 76–81. doi: 10.1016/j.antiviral.2016.11.011 27864075 10.1016/j.antiviral.2016.11.011

[69] García M, Cooper A, Shi W, Bornmann W, Carrion R, Kalman D, et al Productive replication of Ebola virus is regulated by the c-Abl1 tyrosine kinase. Sci Transl Med. 2012;4(123): 123ra24 doi: 10.1126/scitranslmed.3003500 22378924 10.1126/scitranslmed.3003500 PMC4794994

[70] Volchkova VA, Feldmann H, Klenk HD, Volchkov VE. The nonstructural small glycoprotein sGP of Ebola virus is secreted as an antiparallel-orientated homodimer. Virology. 1998;250(2): 408–414. doi: 10.1006/viro.1998.9389 9792851 10.1006/viro.1998.9389

[71] Mehedi M, Falzarano D, Seebach J, Hu X, Carpenter MS, Schnittler H-J, et al A new Ebola virus nonstructural glycoprotein expressed through RNA editing. J Virol. 2011;85(11): 5406–14. doi: 10.1128/JVI.02190-10 21411529 10.1128/JVI.02190-10 PMC3094950

[72] Volchkova VA, Klenk HD, Volchkov VE. Delta-peptide is the carboxy-terminal cleavage fragment of the nonstructural small glycoprotein sGP of Ebola virus. Virology. 1999;265(1): 164–171. doi: 10.1006/viro.1999.0034 10603327 10.1006/viro.1999.0034

[73] Shimojima M, Takada A, Ebihara H, Neumann G, Fujioka K, Irimura T, et al Tyro3 family-mediated cell entry of Ebola and Marburg viruses. J Virol. 2006;80(20): 10109–16. doi: 10.1128/JVI.01157-06 17005688 10.1128/JVI.01157-06 PMC1617303

[74] Lee JE, Saphire EO. Ebolavirus glycoprotein structure and mechanism of entry. Future Virol. 2009;4(6): 621–635. doi: 10.2217/fvl.09.56 20198110 10.2217/fvl.09.56 PMC2829775

[75] Moller-Tank S, Maury W, Herbert A, Kuehne A, Wirchnianski A, Soh T. Ebola Virus Entry: A Curious and Complex Series of Events. PLoS Pathog. 2015;11(4): e1004731 doi: 10.1371/journal.ppat.1004731 25928849 10.1371/journal.ppat.1004731 PMC4415789

[76] Nanbo A, Imai M, Watanabe S, Noda T, Takahashi K, Neumann G, et al Ebolavirus is internalized into host cells via macropinocytosis in a viral glycoprotein-dependent manner. PLoS Pathog. 2010;6(9): e1001121 doi: 10.1371/journal.ppat.1001121 20886108 10.1371/journal.ppat.1001121 PMC2944813

[77] Aleksandrowicz P, Marzi A, Biedenkopf N, Beimforde N, Becker S, Hoenen T, et al Ebola Virus Enters Host Cells by Macropinocytosis and Clathrin-Mediated Endocytosis. J Infect Dis. 2011;204(Suppl 3): S957–S967. doi: 10.1093/infdis/jir326 21987776 10.1093/infdis/jir326 PMC3189988

[78] Mingo RM, Simmons JA, Shoemaker CJ, Nelson EA, Schornberg KL, D’Souza RS, et al Ebola virus and severe acute respiratory syndrome coronavirus display late cell entry kinetics: evidence that transport to NPC1+ endolysosomes is a rate-defining step. J Virol. 2015;89(5): 2931–43. doi: 10.1128/JVI.03398-14 25552710 10.1128/JVI.03398-14 PMC4325712

[79] Simmons JA, D’Souza RS, Ruas M, Galione A, Casanova JE, White JM. Ebolavirus Glycoprotein Directs Fusion through NPC1+ Endolysosomes. J Virol. 2016;90(1): 605–610. doi: 10.1128/JVI.01828-15 26468524 10.1128/JVI.01828-15 PMC4702577

[80] Diehl WE, Lin AE, Grubaugh ND, Carvalho LM, Kim K, Kyawe PP, et al Ebola Virus Glycoprotein with Increased Infectivity Dominated the 2013–2016 Epidemic. Cell. 2016;167(4): 1088–1098. doi: 10.1016/j.cell.2016.10.014 27814506 10.1016/j.cell.2016.10.014 PMC5115602

[81] Urbanowicz RA, McClure CP, Sakuntabhai A, Sall AA, Kobinger G, Müller MA, et al Human Adaptation of Ebola Virus during the West African Outbreak. Cell. 2016;167(4): 1079–1087. doi: 10.1016/j.cell.2016.10.013 27814505 10.1016/j.cell.2016.10.013 PMC5101188

[82] Dietzel E, Schudt G, Krähling V, Matrosovich M, Becker S. Functional Characterization of Adaptive Mutations during the West African Ebola Virus Outbreak. J Virol. 2017;91(2): e01913–16. doi: 10.1128/JVI.01913-16 27847361 10.1128/JVI.01913-16 PMC5215343

[83] Ruedas JB, Ladner J, Ettinger CR, Gummuluru S, Palacios G, Connor JH. Spontaneous mutation at amino acid 544 of the Ebola glycoprotein potentiates virus entry and selection in tissue culture. J Virol. 2017;91(15): e00392–17. doi: 10.1128/JVI.00392-17 28539437 10.1128/JVI.00392-17 PMC5651722

[84] Wang MK, Lim SY, Lee SM, Cunningham JM. Biochemical Basis for Increased Activity of Ebola Glycoprotein in the 2013–2016 Epidemic. Cell Host Microbe. 2017;21(3): 367–375. doi: 10.1016/j.chom.2017.02.002 28238624 10.1016/j.chom.2017.02.002 PMC5735838

[85] Hoffmann M, Crone L, Dietzel E, Paijo J, Gonz?lez-Hern?ndez M, Nehlmeier I, et al A Polymorphism within the Internal Fusion Loop of the Ebola Virus Glycoprotein Modulates Host Cell Entry. J Virol. 2017;91(9): e00177–17. doi: 10.1128/JVI.00177-17 28228590 10.1128/JVI.00177-17 PMC5391465

[86] Groseth A, Marzi A, Hoenen T, Herwig A, Gardner D, Becker S, et al The Ebola virus glycoprotein contributes to but is not sufficient for virulence in vivo. PLoS Pathog. 2012;8(8): e1002847 doi: 10.1371/journal.ppat.1002847 22876185 10.1371/journal.ppat.1002847 PMC3410889

[87] Yang ZY, Duckers HJ, Sullivan NJ, Sanchez A, Nabel EG, Nabel GJ. Identification of the Ebola virus glycoprotein as the main viral determinant of vascular cell cytotoxicity and injury. Nat Med. 2000;6(8): 886–889. doi: 10.1038/78645 10932225 10.1038/78645

[88] Ray RB, Basu A, Steele R, Beyene A, McHowat J, Meyer K, et al Ebola virus glycoprotein-mediated anoikis of primary human cardiac microvascular endothelial cells. Virology. 2004;321(2): 181–188. doi: 10.1016/j.virol.2003.12.014 15051379 10.1016/j.virol.2003.12.014

[89] Sullivan NJ, Peterson M, Yang Z, Kong W, Duckers H, Nabel E, et al Ebola virus glycoprotein toxicity is mediated by a dynamin-dependent protein-trafficking pathway. J Virol. 2005;79(1): 547–53. doi: 10.1128/JVI.79.1.547-553.2005 15596847 10.1128/JVI.79.1.547-553.2005 PMC538691

[90] Zampieri CA, Fortin J-F, Nolan GP, Nabel GJ. The ERK mitogen-activated protein kinase pathway contributes to Ebola virus glycoprotein-induced cytotoxicity. J Virol. 2007;81(3): 1230–40. doi: 10.1128/JVI.01586-06 17108034 10.1128/JVI.01586-06 PMC1797502

[91] Gallaher lliam, Garry R. Modeling of the Ebola Virus Delta Peptide Reveals a Potential Lytic Sequence Motif. Viruses. 2015;7(1): 285–305. doi: 10.3390/v7010285 25609303 10.3390/v7010285 PMC4306839

[92] He J, Melnik LI, Komin A, Wiedman G, Fuselier T, Morris CF, et al Ebola Virus Delta Peptide is a Viroporin. J Virol. 2017;91(16): e00438–17. doi: 10.1128/JVI.00438-17 28539454 10.1128/JVI.00438-17 PMC5533898

[93] Alazard-Dany N, Volchkova V, Reynard O, Carbonnelle C, Dolnik O, Ottmann M, et al Ebola virus glycoprotein GP is not cytotoxic when expressed constitutively at a moderate level. J Gen Virol. 2006;87(Pt 5): 1247–1257. doi: 10.1099/vir.0.81361-0 16603527 10.1099/vir.0.81361-0

[94] Mohan GS, Ye L, Li W, Monteiro A, Lin X, Sapkota B, et al Less is more: Ebola virus surface glycoprotein expression levels regulate virus production and infectivity. J Virol. 2015;89(2): 1205–17. doi: 10.1128/JVI.01810-14 25392212 10.1128/JVI.01810-14 PMC4300637

[95] Kaletsky RL, Francica JR, Agrawal-Gamse C, Bates P. Tetherin-mediated restriction of filovirus budding is antagonized by the Ebola glycoprotein. Proc Natl Acad Sci U S A. 2009;106(8): 2886–91. doi: 10.1073/pnas.0811014106 19179289 10.1073/pnas.0811014106 PMC2650360

[96] Lopez LA, Yang SJ, Hauser H, Exline CM, Haworth KG, Oldenburg J, et al Ebola virus glycoprotein counteracts BST-2/Tetherin restriction in a sequence-independent manner that does not require tetherin surface removal. J Virol. 2010;84(14): 7243–55. doi: 10.1128/JVI.02636-09 20444895 10.1128/JVI.02636-09 PMC2898217

[97] Brinkmann C, Nehlmeier I, Walendy-Gnirß K, Nehls J, González Hernández M, Hoffmann M, et al The Tetherin Antagonism of the Ebola Virus Glycoprotein Requires an Intact Receptor-Binding Domain and Can Be Blocked by GP1-Specific Antibodies. J Virol. 2016;90(24): 11075–11086. doi: 10.1128/JVI.01563-16 27707924 10.1128/JVI.01563-16 PMC5126366

[98] Gustin JK, Bai Y, Moses A V, Douglas JL. Ebola Virus Glycoprotein Promotes Enhanced Viral Egress by Preventing Ebola VP40 From Associating With the Host Restriction Factor BST2/Tetherin. J Infect Dis. 2015;212(Suppl 2): S181–90. doi: 10.1093/infdis/jiv125 25821226 10.1093/infdis/jiv125 PMC4564538

[99] Wahl-Jensen VM, Afanasieva TA, Seebach J, Ströher U, Feldmann H, Schnittler H-J. Effects of Ebola virus glycoproteins on endothelial cell activation and barrier function. J Virol. 2005;79(16): 10442–50. doi: 10.1128/JVI.79.16.10442-10450.2005 16051836 10.1128/JVI.79.16.10442-10450.2005 PMC1182673

[100] McElroy AK, Erickson BR, Flietstra TD, Rollin PE, Nichol ST, Towner JS, et al Ebola Hemorrhagic Fever: Novel Biomarker Correlates of Clinical Outcome. J Infect Dis. 2014;210(4): 558–566. doi: 10.1093/infdis/jiu088 24526742 10.1093/infdis/jiu088 PMC4172044

[101] Dolnik O, Volchkova V, Garten W, Carbonnelle C, Becker S, Kahnt J, et al Ectodomain shedding of the glycoprotein GP of Ebola virus. EMBO J. 2004;23(10): 2175–2184. doi: 10.1038/sj.emboj.7600219 15103332 10.1038/sj.emboj.7600219 PMC424403

[102] Escudero-Pérez B, Volchkova VA, Dolnik O, Lawrence P, Volchkov VE. Shed GP of Ebola Virus Triggers Immune Activation and Increased Vascular Permeability. PLoS Pathog. 2014;10(11): e1004509 doi: 10.1371/journal.ppat.1004509 25412102 10.1371/journal.ppat.1004509 PMC4239094

[103] Olejnik J, Forero A, Deflubé LR, Hume AJ, Manhart WA, Nishida A, et al Ebolaviruses associated with differential pathogenicity induce distinct host responses in human macrophages. J Virol. 2017;91(11): e00179–17. doi: 10.1128/JVI.00179-17 28331091 10.1128/JVI.00179-17 PMC5432886

[104] Iampietro M, Younan P, Nishida A, Dutta M, Lubaki NM, Santos RI, et al Ebola virus glycoprotein directly triggers T lymphocyte death despite of the lack of infection. Thomas PG, editor. PLoS Pathog. 2017;13(5): e1006397 doi: 10.1371/journal.ppat.1006397 28542576 10.1371/journal.ppat.1006397 PMC5456411

[105] Zhao D, Han X, Zheng X, Wang H, Yang Z, Liu D, et al The Myeloid LSECtin Is a DAP12-Coupled Receptor That Is Crucial for Inflammatory Response Induced by Ebola Virus Glycoprotein. PLoS Pathog. 2016;12(3): e1005542 doi: 10.1371/journal.ppat.1005542 26943817 10.1371/journal.ppat.1005487 PMC4778874

[106] Mohan GS, Li W, Ye L, Compans RW, Yang C. Antigenic Subversion: A Novel Mechanism of Host Immune Evasion by Ebola Virus. Basler CF, editor. PLoS Pathog. 2012;8(12): e1003065 doi: 10.1371/journal.ppat.1003065 23271969 10.1371/journal.ppat.1003065 PMC3521666

[107] Pallesen J, Murin CD, de Val N, Cottrell CA, Hastie KM, Turner HL, et al Structures of Ebola virus GP and sGP in complex with therapeutic antibodies. Nat Microbiol. 2016;1(9): 16128 doi: 10.1038/nmicrobiol.2016.128 27562261 10.1038/nmicrobiol.2016.128 PMC5003320

[108] Volchkov VE, Becker S, Volchkova VA, Ternovoj VA, Kotov AN, Netesov SV, et al GP mRNA of Ebola Virus Is Edited by the Ebola Virus Polymerase and by T7 and Vaccinia Virus Polymerases. Virology. 1995;214(2): 421–430. doi: 10.1006/viro.1995.0052 8553543 10.1006/viro.1995.0052

[109] Volchkova VA, Dolnik O, Martinez MJ, Reynard O, Volchkov VE. Genomic RNA Editing and Its Impact on Ebola Virus Adaptation During Serial Passages in Cell Culture and Infection of Guinea Pigs. J Infect Dis. 2011;204(Suppl 3): S941–S946. doi: 10.1093/infdis/jir321 21987773 10.1093/infdis/jir321

